# Estimating the potential impact of the UK government’s sugar reduction programme on child and adult health: modelling study

**DOI:** 10.1136/bmj.l1417

**Published:** 2019-04-17

**Authors:** Ben Amies-Cull, Adam D M Briggs, Peter Scarborough

**Affiliations:** 1 Centre for Population Approaches to Non-Communicable Disease Prevention, Big Data Institute, University of Oxford, Headington, Oxford OX3 7FZ, UK; 2 Centre for Primary Care, University of Manchester, Manchester, UK; 3 National Institute of Health Research Oxford Biomedical Research Centre, Oxford University Hospitals NHS Foundation Trust, Headington, Oxford, UK

## Abstract

**Objective:**

To estimate the impact of the UK government’s sugar reduction programme on child and adult obesity, adult disease burden, and healthcare costs.

**Design:**

Modelling study.

**Setting:**

Simulated scenario based on National Diet and Nutrition Survey waves 5 and 6, England.

**Participants:**

1508 survey respondents were used to model weight change among the population of England aged 4-80 years.

**Main outcome measures:**

Calorie change, weight change, and body mass index change were estimated for children and adults. Impact on non-communicable disease incidence, quality adjusted life years, and healthcare costs were estimated for adults. Changes to disease burden were modelled with the PRIMEtime-CE Model, based on the 2014 population in England aged 18-80.

**Results:**

If the sugar reduction programme was achieved in its entirety and resulted in the planned sugar reduction, then the calorie reduction was estimated to be 25 kcal/day (1 kcal=4.18 kJ=0.00418 MJ) for 4-10 year olds (95% confidence interval 23 to 26), 25 kcal/day (24 to 28) for 11-18 year olds, and 19 kcal/day (17 to 20) for adults. The reduction in obesity could represent 5.5% of the baseline obese population of 4-10 year olds, 2.2% of obese 11-18 year olds, and 5.5% of obese 19-80 year olds. A modelled 51 729 quality adjusted life years (95% uncertainty interval 45 768 to 57 242) were saved over 10 years, including 154 550 (132 623 to 174 604) cases of diabetes and relating to a net healthcare saving of £285.8m (€332.5m, $373.5m; £249.7m to £319.8m).

**Conclusions:**

The UK government’s sugar reduction programme could reduce the burden of obesity and obesity related disease, provided that reductions in sugar levels and portion sizes do not prompt unanticipated changes in eating patterns or product formulation.

## Introduction

Childhood obesity affects 10% of reception class children (aged 4-5) and 20% of year six pupils (aged 11-12) in the United Kingdom.^1^ As part of its Childhood Obesity Plan, the UK government published its report, *Childhood Obesity: A Plan for Action* in August 2016.^2^ This report set a target to reduce children’s sugar consumption by working with food manufacturers to reduce the sugar content of certain high sugar products by 20%, by 2020. This target included food categories that, together with sugary drinks, make up 50% of children’s sugar intake, such as cereals, confectionary, and morning goods (eg, waffles, pancakes). Public Health England (the government body responsible for protecting and improving health in England) proposed to reduce children’s sugar intake by three mechanisms: reformulation of products to contain less sugar, reducing product size, and rebalancing sales weighting (shifting sales from high sugar products to low sugar alternatives).^3^

Sugar in this case is in accordance with the Scientific Advisory Committee on Nutrition’s definition of “free sugars,” that is, all added sugars plus those naturally present in fruit juice, syrups, and honey, but excluding the naturally present sugars in fresh fruit and vegetables or dairy.^4^ The 20% target therefore allows for small amounts of natural sugars to be excluded from the target.^3^ Adults inevitably consume many of the same foods as children, so the programme could have important overlapping health effects for adults. Adult obesity is a health problem in its own right: the proportion of obese adults has risen from 15% in 1993 to 26% in 2016.^1^

The sugar reduction programme follows the Food Standard Agency’s successful approach that reduced average salt intake by 11% between 2003 and 2011, forecasted to prevent 9000 cardiovascular deaths and 9000 non-fatal cardiovascular events per year.^5^ Public Health England has not estimated the potential health effects of the sugar reduction programe; modelling of the UK sugary drinks industry levy (sugary drinks tax)^6^ estimated that it could reduce obesity by 0.9% and prevent 20 000 cases of diabetes per year.

Given the rising burden of obesity related disease across developed and developing countries, sugar has become a key public health target. Sugary drinks taxes have been introduced in several countries including Mexico, France, and Hungary.^6^ The aim of this study was to estimate the potential impact of the UK government’s sugar reduction programme on child and adult obesity, associated health benefits, and healthcare costs.

## Methods

### Modelled scenario

We modelled a scenario estimating the potential changes in weight and body mass index achieved by the UK government’s sugar reduction programme for children and adults. Health modelling was performed, representing a scenario where the programme has been successfully implemented with no unintended consumer or industry behaviour limiting the intended sugar reduction, and the calorie reduction led to weight decrease across the population. Diets were assumed not to change over time. As Public Health England only has remit over England, the rest of the UK was excluded. Respondents aged over 80 were excluded owing to low numbers in the dataset (the National Diet and Nutrition Survey).

### Calorie change and weight modelling

Individual level data on food consumption and foods’ nutritional content was taken from years 5 and 6 of the National Diet and Nutrition Survey (years 2012-13 and 2013-14), providing a baseline diet to calculate age and sex specific changes in calorie intake under the programme. We assumed that food consumption behaviour would not be affected (that is, baseline diet would remain unchanged) apart from a reduction in portion size or sugar content of food items. We assumed no unintended responses to product changes, such as individuals substituting foods because of differences in taste or manufacturers changing non-targeted nutrients (eg, salt).

Foods included in the programme were identified in the National Diet and Nutrition Survey food composition table by food category code (eg, breakfast cereals) and subfood group (eg, porridge oats). For the scenario analysis, either the quantity of sugar in each product was reduced by 20% (for products in the reformulation or sales weighting categories) or total product size was reduced by 20% (for the portion size category). Table 1 summarises which mechanism(s) will mainly be used for each food category. A small allowance for natural sugars in dairy and dried fruit was accounted for, in line with Public Health England’s guidelines, by removing the allowance from total product sugar before the 20% reduction was made, and then adding the allowance back to the reduced sugar component. If more than one sugar reduction mechanism was being used, it was assumed that each mechanism was responsible for equal amounts of sugar reduction, with the total reduction remaining at 20%. Each food item’s new calorie content was then applied to individual diets to create the sugar reduction scenario.

**Table 1:** Sugar reduction mechanism for each food category in the intervention (adapted from the UK government’s sugar reduction programme^3^)

Category description	Mechanism of most relevance to category		
	Reformulation	Portion size	Shifting sales weighting
Breakfast cereals	X		X
Yogurts	X	X	X
Biscuits	X	X	X
Cakes	X	X	
Morning goods	X	X	
Puddings	X	X	
Ice cream, lollies, and sorbets	X	X	
Chocolate confectionery		X	
Sweet confectionery		X	
Sweet spreads and sauces	X		

We used the difference in calorie intake between baseline and the scenario to estimate individual level weight loss, from which we could calculate mean age and sex specific weight loss.

### Weight loss

For children, we estimated weight loss a method adapted from a paper modelling the potential health benefits of the UK soft drinks industry levy.^7^ A meta-analysis of two randomised controlled trials suggested that 0.041 kg of weight was gained for each additional gram of sugar consumed per day for two years.^8^ ^9^ For example, a child who consumed 10 g less of sugar each day under the sugar reduction programme would be estimated to put on 0.41 kg less in weight after two years. These studies were used because they are the only randomised trials quantifying the association between body weight and sugar consumption in children. Because these studies did not include non-sugar calories and child weight modelling is more limited than adult methods, it was not possible to include non-sugar calories in children’s weight change calculations.

We estimated adult weight loss using the Christiansen and Garby method. This method converts changes in the ratio of calories consumed to calories expended into changes in body weight, based on principles of energy conservation (involving measured data on energy expenditure of fat and lean tissue and composition of tissue loss or growth during weight change).^10^ Physical activity data were derived from the National Diet and Nutrition Survey (using code from the Cambridge University Epidemiology Unit (personal communication), which derived the physical activity variables) and were assumed to remain unchanged.

### Estimating change in body mass index

Change in weight was used to calculate change in body mass index, including a small proportion of self reported data (see supplementary material). For children, we used age and sex specific reference cut-off values from the World Health Organization to categorise body mass index as underweight (lighter than two standard deviations below the median), normal (between two standard deviations below the median and one standard deviation above the median), overweight (between one and two standard deviations above the median), and obese (more than two standard deviations above the median).^11^ These data include only children aged 5 and older, so we used the same body mass index cut-off values for 4 year olds as 5 year olds, because these are essentially the same on UK growth charts.^12^ Adult body mass index was categorised as underweight (<18.5), normal (≥18.5 to <25.0), overweight (≥25.0 to <30.0), obese (≥30.0 to <35), and very obese (≥35.0).

### Self reported data, missing data, and imputation

There were missing data for physical activity, height, and weight, which were imputed separately by age (separately for 4-18 and 19-80 year olds) and sex, using age and daily calorie intake as predictor variables for linear regression models, with likelihood ratio tests used to determine best fit. We introduced random variation to imputed data using a random number generator with a normal distribution and a standard deviation equal to the sex specific residuals of the variable plotted against its predictors. Self reported data were included. The percentages of missing and self reported data for each variable are summarised in the supplementary material.

### Chronic disease impact modelling

We estimated the 10 year impact of decreased adult body weight on chronic disease for the 2014 population aged 18-80 in England using PRIMEtime-CE, a cohort chronic disease multistate lifetable model. The structures and assumptions that underpin such models are described in detail elsewhere,^13^ as are details on the PRIMEtime-CE model itself.^14^ Briefly, the model is a set of linked, multi-state, life table models where the population is divided into healthy, diseased, and dead states with transition parameters controlling flow between these states, which are informed by epidemiological data on incidence and case fatality. Each of these multi-state, life table models follows the Markovian assumption: that individuals are not identified in the modelling process and so the model has no memory (that is, transition rates between states are not affected by the length of time spent in them). Similar to other proportional multistate life table models, PRIMEtime-CE calculates multi-morbidity by combining prevalence rates multiplicatively, assuming that the prevalence of one disease does not alter any other. The exception to this rule is diabetes, which operates both as a disease endpoint and as a risk factor for other diseases in PRIMEtime-CE.^14^

We used change in adult weight to estimate the UK government sugar reduction programme’s impact on morbidity and mortality from cardiovascular disease, stroke, diabetes, breast cancer, colorectal cancer, cirrhosis, pancreatic cancer, kidney cancer, and liver cancer. In scenario analyses, relative risks between body mass index and these disease outcomes (derived from the literature) are used to estimate population impact fractions,^15^ which are used to alter disease incidence. We estimated associated quality adjusted life years (QALYs) using utility weights, and healthcare costs using NHS England programme budgeting costs and specialised services expenditure. We accounted for future costs to healthcare costs from diseases not included in the model but arising due to longevity. This modelling process has been described previously.^14^ ^16^ For the health modelling step, weight change in 18 year olds was recalculated by the adult method to keep consistency with the rest of the modelled sample. Disease and cost impacts were not estimated for children. The choice of the 10 year time horizon was based on previous work with stakeholders that identified this period as the most pertinent timeframe to consider.^17^

We prepared the dataset Stata 12 and executed the PRIMEtime-CE Model with 2000 runs of Monte Carlo analysis using Microsoft Excel 2016 and Ersatz 1.35 (Epigear International 2017). The Monte Carlo analysis was used to generate 95% uncertainty intervals, representing the range from the 2.5th to 97.5th centiles of the Monte Carlo analysis outcomes. The parameters that were allowed to vary were relative risks between body mass index and disease outcomes, hospital costs, and utility values.

### Sensitivity analyses

We performed four sensitivity analyses. Firstly, we simulated situations where one mechanism or another failed to reduce calorie consumption as planned. Secondly, we repeated health modelling using three year rolling averages of body mass index data over the age range to reduce the volatility of small numbers in each age group. Thirdly, we excluded individuals whose physical activity, height, or weight was imputed or self reported. Finally, we modelled extended time horizons of 30 and 100 years (a “lifetime”).

### Patient and public involvement

No patients were involved in deciding the research question, study design, outcome measures, or in the interpretation of results. There are no plans to disseminate results of the research to patient groups. This work uses data provided by participants and this work would not have been possible without access to these data. The authors recognise and value the role of participants’ data, securely accessed and stored, both in underpinning and leading to improvements in research and care.

## Results

### Sample

A total of 1508 participants in the National Diet and Nutrition Survey waves 5 and 6 completed at least three diary days, lived in England, and were aged 4-80 years. Calorie, body weight, and body mass index data had sampling weights applied. Table 2 shows a summary of the estimated impact of the sugar reduction programme on energy intake, body weight, body mass index, and body mass index category, by participants’ age and sex. If the sugar reduction programme was achieved in its entirety and resulted in the planned sugar reduction, then the calorie reduction was estimated to be 25 kcal/day (1 kcal=4.18 kJ=0.00418 MJ) for 4-10 year olds (95% confidence interval 23 to 26), 25 kcal/day (24 to 28) for 11-18 year olds, and 19 kcal/day (17 to 20) for adults.

**Table 2:** Estimated impact of the UK government’s sugar reduction programme on calorie consumption and weight

Outcome measures	Age and sex of participants							
	4-10 years			11-18 years			19-80 years	
	Male	Female		Male	Female		Male	Female
Sugar reduction (kcal/day; 95% CI)	25.7 (23.6 to 27.8)	23.5 (21.6 to 25.5)		28.2 (25.2 to 31.3)	22.4 (20.3 to 24.6)		20.7 (18.0 to 22.9)	17.0 (15.4 to 18.6)
Sugar reduction as proportion of baseline sugar intake (%; 95% CI)	7.0 (6.6 to 7.4)	7.2 (6.8 to 7.6)		6.4 (5.9 to 6.9)	6.3 (5.9 to 6.8)		5.0 (4.5 to 5.1)	5.0 (4.7 to 5.2)
Sugar reduction as proportion of baseline total calories (%; 95% CI)	1.6 (1.5 to 1.8)	1.6 (1.5 to 1.8)		1.4 (1.3 to 1.5)	1.4 (1.3 to 1.5)		0.9 (0.9 to 1.0)	1.0 (1.0 to 1.1)
Calories reduced from non-sugar nutrients (kcal/day; 95% CI)	—	—		—	—		5.6 (4.4 to 6.3)	4.1 (3.5 to 4.7)
Total sugar and non-sugar calorie reduction as a proportion of total baseline calories (%; 95% CI)	—	—		—	—		1.2 (1.1 to 1.3)	1.3 (1.2 to 1.4)
Weight change based on sugar (kg; 95% CI)	0.28 (0.26 to 0.30)	0.26 (0.24 to 0.28)		0.31 (0.28 to 0.34)	0.25 (0.22 to 0.27)		—	—
Weight change based on sugar and non-sugar (kg; 95% CI)	—	—		—	—		1.51 (1.37 to 1.65)	1.77 (1.64 to 1.90)
Corresponding change in body mass index (95% CI)	0.18 (0.17 to 0.20)	0.17 (0.16 to 0.19)		0.11 (0.10 to 0.12)	0.10 (0.08 to 0.12)		0.51 (0.46 to 0.55)	0.67 (0.63 to 0.71)

1 kcal=4.18 kJ=0.00418 MJ.

Calories from non-sugar nutrients were not included in calculations for weight change in children because this method was derived from evidence using changes to sugar intake^8^ ^9^

### Sugar consumption

Achieving the programme’s sugar reduction targets could result in the average energy consumption in 4-10 year-olds from sugar falling by 23.5 kcal/day (95% confidence interval 21.6 to 25.5) for girls and 25.7 kcal/day (23.6 to 27.8) for boys, equating to 7.2% (6.8% to 7.6%) and 7.0% (6% to 7.4%) less sugar calories per day, respectively. For 11-18 year olds, girls were estimated to consume 22.4 kcal/day (20.3 to 24.6) less from sugar and boys estimated to consume 28.2 kcal/day less (25.2 to 31.3), or an equivalent of 6.3% (5.9% to 6.8%) and 6.4% (5.9% to 6.9%) of baseline sugar calories, respectively. The effect on adult (19-80 years) sugar consumption was smaller, at an average of 17.0 kcal/day (15.4 to 18.6) or 5.0% (4.7% to 5.2%) of baseline sugar calories for women, and 20.7 kcal/day (18.0 to 22.9) or 5.0% (4.5% to 5.1%) for men. Non-sugar calories lost in portion reduction among adults contributed an average additional 4.1 kcal/day for women and 5.6 kcal/day for men, although these were highly skewed, with a median of 0.0 kcal/day for both sexes and interquartile range of 0.0-5.8 kcal/day for women and 0.0-6.7 kcal/day for men.

### Effect on weight

The mean difference in weight for 4-10 year olds was 0.26 kg (95% confidence interval 0.24 to 0.28) for girls and 0.28 kg (0.26 to 0.30) for boys. These differences equated to a reduction in body mass index of 0.17 (0.16 to 0.19) for girls and 0.18 (0.17 to 0.20) for boys. For 11-18 year olds, girls were estimated to lose 0.25 kg (0.22 to 0.27) and boys 0.31 kg (0.28 to 0.34), equating to a change in body mass index of 0.10 (0.08 to 0.12) and 0.11 (0.10 to 0.12), respectively. The proportion of 4-10 year olds classified as overweight fell from 26.0% to 22.5% for girls and from 18.9% to 16.6% for boys. In the 4-10 year old group, the proportion of obese girls remained unchanged but the proportion of obese boys fell by 1.2% to 9.7%. For 11-18 year olds, the proportion of overweight girls fell from 19.2% to 18.6%; the proportion of overweight boys remained unchanged at 13.6%. The proportion of obese 11-18-year-old girls also stayed the same at 9%, and the proportion of obese boys fell slightly from 13.6% to 13.0%. In total, the obese population was estimated to shrink by 5.5% of baseline cases for 4-10 year olds (0.6 percentage points), 2.2% for 11-18 year olds (0.3 percentage points), and 5.5% for 19-80 year olds (2.3 percentage points).

**Fig 1:**
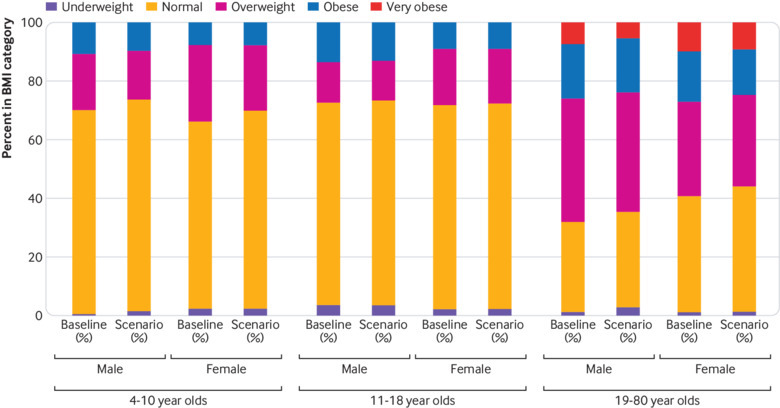
Body mass index category before and after intervention for each participant age group. Intervention based on the UK government’s sugar reduction programme

For 19-80 year-olds, the weight change for women was an average of 1.77 kg (95% confidence interval 1.64 to 1.90), representing a reduction of 0.67 (0.63 to 0.71) in body mass index. Men had an average weight loss of 1.51 kg (1.37 to 1.65) and a reduction of 0.51 (0.46 to 0.55) in body mass index. This weight loss translated into a fall in the proportion of overweight 19-80 year old women from 32.1% to 30.8%, while the proportion of obese women fell from 17.3% to 15.4% and very obese women from 9.8% to 9.0%. The proportion of overweight men fell from 42.3% to 40.8%, obese men from 18.4% to 17.3%, and very obese men from 7.3% to 5.4%. These changes to the distribution of body mass index in adults and children are shown in figure 1.

### Disease burden impact and healthcare costs savings

The modelling process estimated that the increased impact on women’s body mass index was likely to be realised as greater health benefits for women over men. Over 10 years, a total of 27 855 QALYs could be saved for women (95% uncertainty interval 24 573 to 30 873) and 23 874 QALYs for men (21 194 to 26 369). By far the biggest impact on disease incidence was for diabetes, with 154 550 fewer cases over 10 years (89 571 for women (101 081 to 76 925) and 64 979 for men (55 698 to 73 523)). Net incidence of lung cancer and gastric cancer slightly increased owing to increased longevity. The full breakdown of impact on disease incidence is summarised in table 3. The cost estimation process projected a net saving to healthcare of £285.8m (€333m; $374.2m) over 10 years, which comprises £161.6m from women (£141.6m to £180.6m) and £124.2m from men (£108.1m to £139.2m).

**Table 3:** Estimated impact of the UK government’s sugar reduction programme on quality adjusted life years (QALYs), disease burden, and healthcare costs over 10 years

Characteristic	Mean (2.5th-97.5th centile; uncertainty interval)	
	Male population	Female population
QALYs (No)	23 874 (21 194 to 26 369)	27 855 (24 573 to 30 873)
NHS costs (£m)*	−£124.2 (−£139.2 to −£108.1)	−£161.6 (−£180.6 to −£141.6)
Disease burden (No of cases)		
Cardiovascular disease	−1591 (−1742 to −1433)	−1920 (−2118 to −1733)
Stroke	−881 (−987 to −771)	−1741 (−1947 to −1524)
Diabetes	−64 979 (−73 523 to −55 698)	−89 571 (−101 081 to −76 925)
Breast cancer	0	−2872 (−3759 to −1972)
Colorectal cancer	−4220 (−4845 to −3575)	−1573 (−2251 to −891)
Lung cancer	21 (18 to 23)	11 (10 to 12)
Stomach cancer	3 (3 to 4)	1 (1 to 2)
Cirrhosis	−2982 (−3649 to −2248)	−2620 (−3216 to −1961)
Pancreas cancer	−251 (−335 to −166)	−297 (−397 to −196)
Kidney cancer	−863 (−1152 to −562)	−865 (−1060 to −672)
Liver cancer	−643 (−873 to −402)	−425 (−576 to −265)

* £1=€1.17; $1.31.

### Sensitivity analyses

The reformulation mechanism was estimated to account for 40% of the calorie reduction in 4-10 year olds (9.8 kcal/day), 34% in 11-18 year olds (8.6 kcal/day), and 37% in 19-80 year olds (7.0 kcal/day). Corresponding figures were 43% (10.6 kcal/day), 51% (12.9 kcal/day), and 46% (8.6 kcal/day) for portion reduction, respectively; and 17% (4.2 kcal/day), 16% (4.0 kcal/day), and 17% (3.2 kcal/day) for sales weighting, respectively. For 19-80 year olds, this proportion included non-sugar calories removed in portion size reduction, because these can be included in the method for adult weight change. 

As shown in table 4, the failure of one of the mechanisms (reformulation, portion reduction, or sales weighting) to successfully reduce calorie intake attenuated the impact on disease prevention; this attenuation was roughly proportionate to the difference in calorie reduction. The 30 year time horizon estimated much greater impacts on health than the 10 year horizon, with over seven times more QALYs saved (378 002 QALYs) and over five times as much in NHS costs (£1.56bn). Similarly, the lifetime horizon anticipated 16 times more QALYs spared than the 10 year horizon (839 274 QALYs) and nine times the NHS costs (£2.71bn) over 100 years. Over these increased time periods, the increased effect of the intervention on women’s body mass index translated into slightly greater impacts on women’s non-communicable disease burden and healthcare costs. For instance, the intervention’s impact on total QALY burden for women was 16% greater than for men over 10 years, 18% over 30 years, and 25% over 100 years. The remainder of the sensitivity analyses identified very small differences from the main analysis. 

**Table 4:** Summary outputs of sensitivity analyses

Characteristic	30 year duration	Lifetime duration	Verified height and weight only	Including over 80s	Body mass index, 3 year average	Excluding reformulation	Excluding portion reduction	Excluding sales weighting
**Disease burden (No of cases)**								
Cardiovascular disease								
Male	−7363	−17 377	−1630	−2007	−1630	−880	−959	−1387
Female	−9552	−27 134	−1898	−2300	−1966	−1098	−1151	−1663
Stroke								
Male	−5272	−14 380	−915	−955	−921	−500	−526	−771
Female	−11 144	−34 634	−1759	−1770	−1796	−993	−1056	−1511
Diabetes								
Male	−205 572	−315 785	−67 885	−68 353	−66 991	−37 135	−40 587	−56 794
Female	−283 734	−445 185	−91 391	−89 757	−92 542	−50 280	−57 008	−78 395
Breast cancer								
Male	0	0	0	0	0	0	0	0
Female	−8576	−15 514	−2811	−3616	−2975	−1709	−1667	−2431
Colorectal cancer								
Male	−12 016	−20 021	−4277	−5971	−4308	−2392	−2438	−3662
Female	−4446	−7431	−1556	−2002	−1616	−908	−939	−1362
Lung cancer								
Male	358	1376	21	29	22	12	12	18
Female	182	875	11	14	12	7	7	10
Stomach cancer								
Male	62	268	3	5	3	2	2	3
Female	29	157	1	2	2	1	1	1
Cirrhosis								
Male	−8005	−11 745	−3098	−3120	−3061	−1684	−1841	−2599
Female	−7072	−10 685	−2674	−2620	−2718	−1472	−1642	−2280
Pancreatic cancer								
Male	−722	−1148	−253	−412	−256	−144	−143	−219
Female	−868	−1456	−287	−422	−301	−172	−173	−255
Kidney cancer								
Male	−2430	−3913	−877	−1164	−885	−484	−511	−755
Female	−2495	−4249	−845	−1057	−880	−495	−515	−750
Liver cancer								
Male	−2430	−3913	−877	−1164	−885	−484	−511	−755
Female	−1454	−2619	−404	−620	−434	−253	−249	−373
**Quality adjusted life years (No)**								
Male	173 310	372 798	24 559	26 003	23 795	13 114	15 083	20 902
Female	204 692	466 476	28 554	28 872	28 577	15 426	17 666	24 342
**NHS costs (£m)**								
Male	−£631	−£1027	−£128	−£131	−£123	−£68	−£79	−£109
Female	−£932	−£1684	−£166	−£165	−£166	−£89	−£103	−£141

In addition to the main sensitivity analyses, the two weight-change methods were compared for 18 year-olds. For the mean calorie reduction in male participants of 19.9 kcal, the children’s method estimated a 0.82 kg weight loss, versus 1.5 kg by the adult method.

## Discussion

According to our estimates, the UK government’s sugar reduction programme could reduce obesity among 4-10 year olds by 5.5%, 11-18 year olds by 2.2%, and 19-80 year olds by 5.5%. But for this to happen, the programme’s goals need to be met in their entirety and without its introduction leading to unintended changes in consumer or industry behaviour.

The programme could also achieve reductions in obesity related disease in adults. The largest impact was on type 2 diabetes, with 155 000 fewer cases over 10 years, representing about 7% of baseline incidence on an annualised basis. By comparison, an evaluation of the UK’s soft drinks industry levy estimated (via an unrelated method) 20 000 fewer cases of diabetes per year owing to a 10 kcal/day reduction in sugar intake (compared with a 25 kcal/day reduction in this study).^7^ Savings can also be compared with Public Health England’s methods for estimating the impact of the ambitious goal of sugar intake falling to 5% of daily calories.^18^ ^19^ These methods estimated a £576m saving per year from cutting 11% of daily calories (equivalent to £52m for each 1% calorie reduction), compared with our estimate of £28.6m per year for a 1% calorie reduction. To put these figures in context, Public Health England estimate that healthcare costs of obesity related disease are £5.1bn annually,^2^ and the sugary drinks tax is forecast to generate £275m for the Treasury in 2018.^20^ Extending the time horizon to 30 or 100 years increased the anticipated health and healthcare cost benefits, with an increasing QALY benefit over time.

The potential health benefits of the programme could be lost if any of the mechanisms being used to reduce sugar consumption fail to have the intended effect. The success of the programme relies on how consumers and industry respond. Early evidence indicates that industry has not met the ambitious target of achieving a quarter of the total reduction in the first year of the programme.^21^ Industry might also adjust portion sizes to contribute to their targets, for example, by rebranding products to a “sharing” size.^22^ The reformulation approach might also fail if industry replaces sugar calories with other calories or if reformulated products are commercially unsuccessful.

Consumer response is also difficult to anticipate. For example, if portion size is reduced then people could simply eat a larger number of portions, but moderate quality evidence indicates a link between larger portions and increased intake.^23^ There is evidence that people with high sugar diets are more likely to be overweight or obese, possibly because the body fails to effectively detect sugar calories.^24^ For children, strong evidence suggests that sugar reformulation leads to change in weight status, based on randomised studies. No randomised studies have explored product reformulation as a means of sugar reduction and weight management in adults. Therefore, reformulation might not lead to calorie reduction, for example, if people replace sugar calories with other calories. The sensitivity analyses simulating the failure of each mechanism indicate that these uncertainties could be important.

The only randomised trials quantifying the effect of sugar on body weight in children used sugary drinks as the intervention, so we made an assumption that sugar consumed as foods was the same as drinks. However, stronger evidence has indicated a link between sugary drinks and obesity than a link between sugary foods and obesity.^4^ More broadly, the approach of sugar reduction itself needs to be approached judiciously, because the links between sugar and obesity have not yet been conclusively described.^4^ ^25^ ^26^

The sugar reduction programme comes in the context of sugar becoming a key public health target around the world, including sugary drinks taxes in the UK and several other countries. This collaborative approach with industry to reduce sugar consumption is novel.^3^ Relevant to food and health policy, we find that small amounts of dietary change at the individual level are cumulatively important at the scale of the whole population, consistent with evidence for structural interventions being highly effective for improving population health (over individual interventions).^27^ Given the scale of the non-communicable disease epidemic around the world, structural public health approaches such as this could be important in effectively reducing risk.

### Limitations

Firstly, scenario modelling research has perennial issues such as response bias in underlying data, reliance on observational studies to determine associations between risk factors and disease, and an inability to validate results with external datasets. Previous research has quantified under-reporting of calorie intake in the National Diet and Nutrition Survey on average by 11-36% depending on population subgroup, comparable with similar surveys.^28^

In the present study, the methods of estimating weight change for both children and adults had limitations. For 18 year olds, the mean calorie reduction in male participants of 19.9 kcal estimated a 0.82 kg weight loss using the children’s method and a 1.5 kg weight loss using the adult method. This difference indicates that the more simplistic weight change method for children might be conservative, at least for older children. An alternative method of estimating adult weight change developed by Hall and colleagues is considered the gold standard, as it has been validated against double labelled water studies.^29^ ^30^ The Hall method could not be incorporated into modelling owing to the complexity of the model and incompatibility between software languages. However, examples were compared (see supplementary material), identifying a tendency for the Hall method to estimate slightly greater calorie change requirements than the Christiansen and Garby method. These methods have also previously been compared, identifying very small differences in estimated weight change between the methods for the levels of calorie change relevant to this paper.^31^

### Conclusion

Based on our estimates, the UK government’s sugar reduction programme could have a useful effect on child obesity. Since 2006-07, the rate of obesity has remained at 10% in reception age children (aged 4-5) and increased from 18% to 20% for year six pupils (age 11-12).^1^ The reduction in obese children aged 4-10 modelled here could account for a 0.6% reduction in the obesity rate for reception age children. For children aged 11-18, however, the impact of the programme on obesity is likely to be smaller, at around a 0.3% difference.

Cost savings could be made through reduced disease burden in adults. The failure of an approach (portion size reduction, reformulation, or sales weighting) could lead to substantial benefits being lost, in line with the proportion of calories not being removed from people’s diets. Benefits continue to be seen over longer time horizons. These findings imply that the sugar reduction programme could be an effective means of reducing obesity related illness and costs, although targets must be met. As the programme targets child health through foods that children tend to consume, there could be another opportunity to target adult health and diet through foods that are more consumed by adults. Structural approaches to the prevention of non-communicable diseases such as this programme offer great opportunities to reduce disease burden.

**What is already known on this topic**

In March 2017, the UK government set out its plans to work with the food and retail industries to reduce the sugar content of certain food groups, such as cereals and confectionary, by 20% by 2020
Public Health England modelled that meeting a different target of sugar intake being no more than 5% of calorie intake would reduce average calorie intake by 11%, leading to 4700 fewer sugar related deaths per year and a healthcare cost saving of £576m per year
The sugar reduction programme aims to reduce childhood obesity, but its potential health benefits have not yet been estimated

**What this study adds**

Estimates indicate that the sugar reduction programme could reduce the proportion of obese children by 5.5% in those aged 4-10, by 2.2% in those aged 11-18, and by 5.5% in adults
The programme was modelled to reduce calorie intake by 25 kcal/day for children and 19 kcal/day for adults (1 kcal=4.18 kJ=0.00418 MJ); in adults, this intake reduction could lead to 155 000 fewer cases of diabetes, 3500 fewer cases of cardiovascular disease, 5800 fewer cases of colorectal cancer, and a total NHS cost saving of £286m over 10 years
Scenarios simulating the failure of one of the three sugar reduction approaches (reformulation, portion reduction, or sales weighting) found that health benefits could be easily lost

## Web extra

Extra material supplied by authors

**Web appendix:** Supplementary material

## Notes

Contributors: BA-C acts as guarantor; contributed to the study design, literature search, figures, data analysis, and data interpretation; and wrote the first draft of the manuscript. ADMB contributed to the data analysis, data interpretation, and manuscript preparation. PS contributed to the study design, literature search, figures, data analysis, data interpretation, and manuscript preparation. All authors, internal and external, had full access to all of the data (including statistical reports and tables) in the study and can take responsibility for the integrity of the data and the accuracy of the data analysis. The corresponding author attests that all listed authors meet authorship criteria and that no others meeting the criteria have been omitted.

Ethical approval: The terms of the University of Oxford (where the research took place) medical sciences interdivisional research ethics committee were consulted and this proposal did not require ethics approval because it involves only fully anonymised precollected data.

Data sharing: Modelled outcomes data are available from the corresponding author. National Diet and Nutrition Survey data are available with standard conditions of use.

The lead author and guarantor affirm that the manuscript is an honest, accurate, and transparent account of the study being reported; that no important aspects of the study have been omitted; and that there are no discrepancies from the study as originally planned.
