# Supplementary material for: Estimating the potential impact of the UK government’s sugar reduction programme on child and adult health: modelling study
Source: BMJ. 2019 Apr 17;365:l1417. doi: 10.1136/bmj.l1417 (PMC6468887; doi:10.1136/bmj.l1417)
Supplement: Supplementary file 1 — Web appendix: Supplementary material [file amib046887.ww.pdf]

Age group	Variable	Total respondents	Number imputed	Percent imputed	Number self-reported	Percent self-reported
4-10 years	Weight	344	11	3.2	11	3.2
	Height	344	12	3.5	13	3.8
11-18 years	Weight	346	5	1.4	5	1.4
	Height	346	5	1.4	5	1.4
19-80 years	Weight	851	33	3.9	36	4.2
	Height	851	31	3.6	40	4.7
18-80 years	PAL	851	18	2.1	n/a	n/a

S1: Information on quantities of imputed data

Inputs:										Estimated calorie change:		
Age	Sex	Height centile	Height (cm)	Weight centile	Weight (kg)	PA* centile	PA (METs*)	Weight change centile	Weight change (kg)	Christiansen and Garby (kcal)	Hall et al, for weight loss (kcal)	Hall et al, for subsequent weight maintenance (kcal)
40	F	50	163	50	68.6	50	1.4	50	1.5	24	35	27
40	M	90	185	50	83.5	90	2.6	10	0.2	7	15	15
40	F	50	163	50	68.6	50	1.4	90	3.6	58	84	64
70	F	10	153	10	54.3	10	1.9	50	1.5	33	48	44
70	M	50	175	90	105.1	10	1.2	90	3.2	52	63	32

* PA = Physical Activity; METs = Metabolic Equivalents

Methods used for table S2:

Due to the user interface for Hall et al, it is not possible to calculate weight change from calorie change as we did using the Christiansen and Garby method (reference 9). Instead, we were able to compare different cases of the input parameters to the models (height, weight, physical activity level and weight change) at the 10th, 50th and 90th centiles, to demonstrate cases at the centres and extremes of these inputs. These demonstrate that Hall et al generally predicted a slightly greater requirement of calorie reduction for a given weight loss.

References:

Christiansen E, Garby L. Prediction of body weight changes caused by changes in energy balance. Eur J Clin Invest. 2002;32(11):826–830

Hall KD, Sacks G, Chandramohan D, Chow CC, Wang YC, Gortmaker SL, et al. Quantification of the effect of energy imbalance on

bodyweight. The Lancet. 2011;378(9793):826–837
National Institute of Diabetes and Digestive and Kidney Diseases. Body Weight Planner. Available from: https://www.niddk.nih.gov/bwp
